# Evaluation of different landing pages on behavioural engagement with the CARA dashboard: A user research protocol

**DOI:** 10.1186/s12875-024-02420-6

**Published:** 2024-05-20

**Authors:** Nathaly Garzón-Orjuela, Heike Vornhagen, Catherine Blake, Akke Vellinga

**Affiliations:** 1 CARA Network, School of Public Health, Physiotherapy and Sports Science, University College Dublin, Dublin, Ireland; 2 Insight Centre for Data Analytics, University of Galway, Galway, Ireland; 3 School of Public Health, Physiotherapy and Sports Science, University College Dublin, Dublin, Ireland

**Keywords:** Dashboard, User research, Primary health care, General practice

## Abstract

**Background:**

CARA set out to develop a data-visualisation platform to facilitate general practitioners to develop a deeper understanding of their patient population, disease management and prescribing through dashboards. To support the continued use and sustainability of the CARA dashboards, dashboard performance and user engagement have to be optimised. User research places people at the centre of the design process and aims to evaluate the needs, behaviours and attitudes of users to inform the design, development and impact of a product.

**Objective:**

To explore how different initial key messages impact the level of behavioural engagement with a CARA dashboard.

**Methods:**

Participating general practices can upload their practice data for analysis and visualisation in CARA dashboards. Practices will be randomised to one of three different initial landing pages: the full dashboard or one of two key messages: a between comparison (their practice prescribing with the average of all other practices) or within comparison (with practice data of the same month the previous year) with subsequent continuation to the full dashboard. Analysis will determine which of the three landing pages encourages user interaction, as measured by the number of ‘clicks’, ‘viewings’ and ‘sessions’. Dashboard usage data will be collected through Google analytics.

**Discussion:**

This study will provide evidence of behavioural engagement and its metrics during the implementation of the CARA dashboards to optimise and sustain interaction.

**Trial registration:**

ISRCTN32783644 (Registration date: 02/01/2024).

## Background

Most general practices have computerised patient management systems (PMS). However, few PMS provide options to perform data reporting and exploration, audit and feedback, in-depth analysis of aggregated patient data or comparison and benchmarking with other general practices [1, 2]. The use of dashboards and data visualisations is growing in the health sector, in particular since the pandemic, when numerous exemplars were designed to allow fast and easy comparisons [3, 4]. However, these visual analytics processes imply access and integration of data [5] and challenges remain as data is recorded through different, often incompatible systems (data silos) for which project specific, bespoke dashboards are designed [4, 6].

Dashboards are generally launched for one sole purpose, such as research, exploratory, analytical or business. Dashboard are on the whole, designed ‘for’ and not ‘with’ users and allow limited, if any, user interaction or engagement [3, 7, 8]. Once set up, dashboards require continuous input from the developers to maintain their relevance [3], which must be tailored to the end users’ needs. Developers should employ iterative evaluation of dashboard performance during the development and implementation to promote uptake and use of the dashboard [9]. Researchers and developers have to maintain the effectiveness and relevance of the dashboard in terms of health outcomes and user engagement.

User engagement is a complex concept that incorporates emotional, cognitive and behavioural dimensions [10]. Emotional engagement entails the processing of visual, tactile, auditory and interactive cues by the user’s attentional faculties. Cognitive engagement pertains to problem-solving and sensory aspects of the experience, encompassing enjoyment, boredom, curiosity, stimulation of imagination and evoked interest. Behavioural engagement is linked to interactivity and serves as a key element in enhancing the sensory dimension of the experience [10]. Behavioural engagement revolves around the user’s interactions with the product and their responses to its feedback. It includes the frequency and extent of user interactions, along with the time needed to accomplish tasks or engage with content. Interaction serves as a tangible representation of a user’s behavioural engagement with the product, signifying active user participation during these stages. The metrics employed typically involve tracking the number of clicks, pages visited, video durations watched and views of specific content pages [10].

User research combines qualitative and quantitative research methods to evaluate the needs, behaviours and attitudes of users to inform the design, development and impact of a product [11]. For the evaluation of a data dashboard, a process evaluation involves understanding how users interaction with the dashboard (i.e., clicks, views and duration on the interaction), which helps inform the dashboard’s adaptation to maximise user exposure [12]. Monitoring web traffic sources through Google Analytics provides quantitative data on dashboard usage [12, 13] and allows measuring user perceptions or behaviour indirectly [12, 14–16]. Furthermore, these web traffic sources allow developers to estimate and infer the level of behavioural engagement on website platforms. Overall, a strong level of behavioural engagement groups several indicators from Google Analytics, such as high returning users numbers, low bounce rate, high page viewed numbers per session, high mean session duration and lofty goal conversion rates [12, 16, 17].

The CARA project is designed as a sustainable data-sharing platform that can be applied across different PMS to facilitate general practitioners to develop a deeper understanding of their patient population, disease management and prescribing through dashboards [18, 19]. The CARA infrastructure consists of a common data model (to combine data from different PMS), CARAconnect to upload data and CARA dashboards for visualising data. The first exemplar dashboard is focused on antibiotic prescribing and includes automated audit reports, filters (within practice) and between-practice comparisons [18, 19]. The antibiotic Anatomical Therapeutic Chemical code J01 was used for this antibiotic dashboard and categorised into green (preferred – antibiotics are more effective, have fewer side effects and are less likely to lead to resistant infections) and red (non-preferred - antibiotics should not be used in primary care unless absolutely necessary) antibiotics according to Irish national guidelines [20, 21].

To design the CARA dashboard, action design research (ADR) was used, which generates knowledge about how to solve organisational problems in practice. ADR includes four steps (a) problem formulation, (b) building, intervention and evaluation, (c) reflection and learning and (d) formalisation of learning [22, 23]. During the development and testing of the CARA dashboard, it became clear that dashboard performance (i.e. behavioural engagement and interaction) is important for continued use and sustainability of the dashboard. Hence, it is essential to implement user research to quantify how users (practices) interact with the dashboards and how to influence user behaviour. Previous research showed that behavioural interventions based on peer comparison can have a marked effect on the prescribing decisions of health professionals [24–26]. This study will implement user research to explore how different initial key messages impact the level of behavioural engagement with the CARA dashboard.

## Method

### Study design

**Fig. 1:**
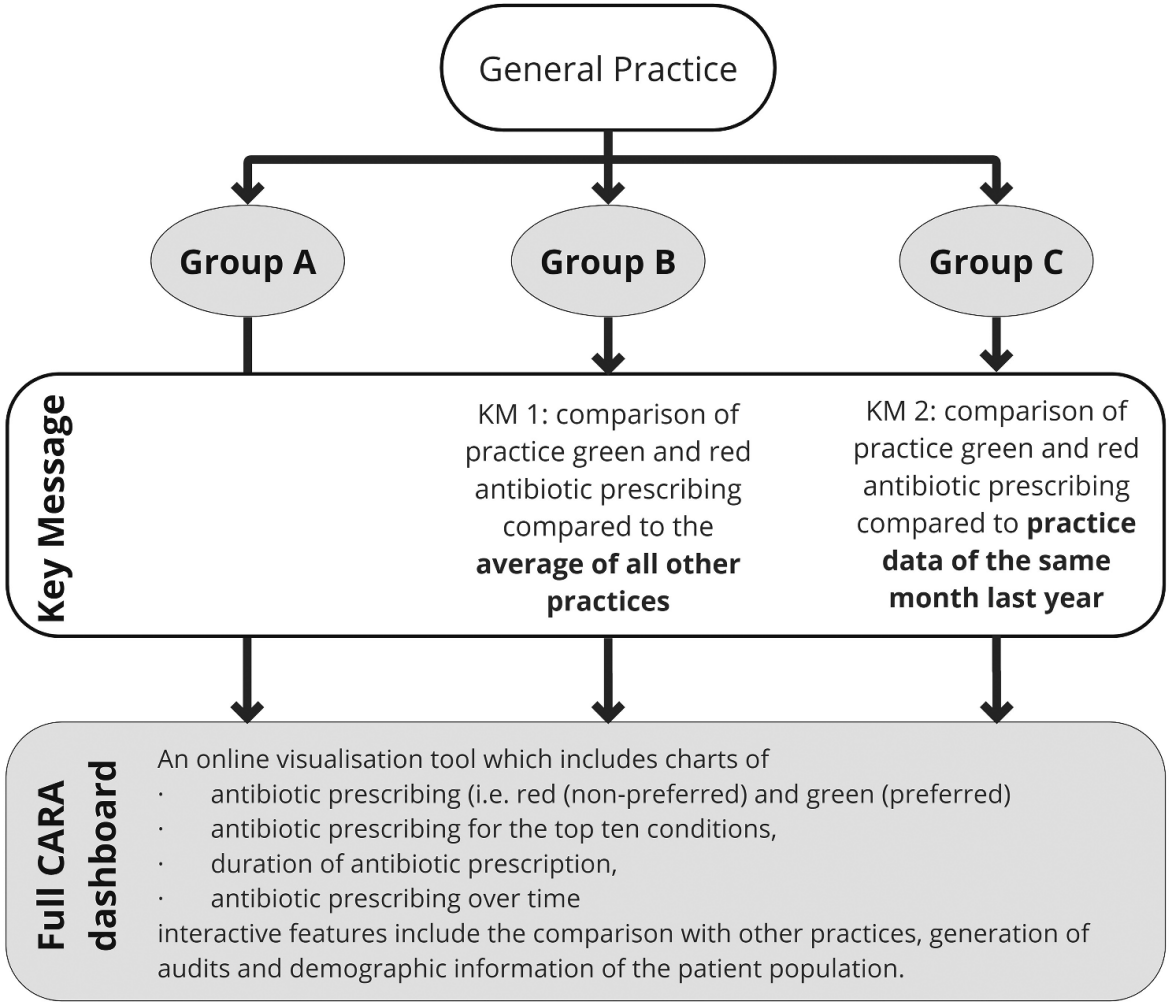
Details of the three different initial landing pages (groups)

**Fig. 2:**
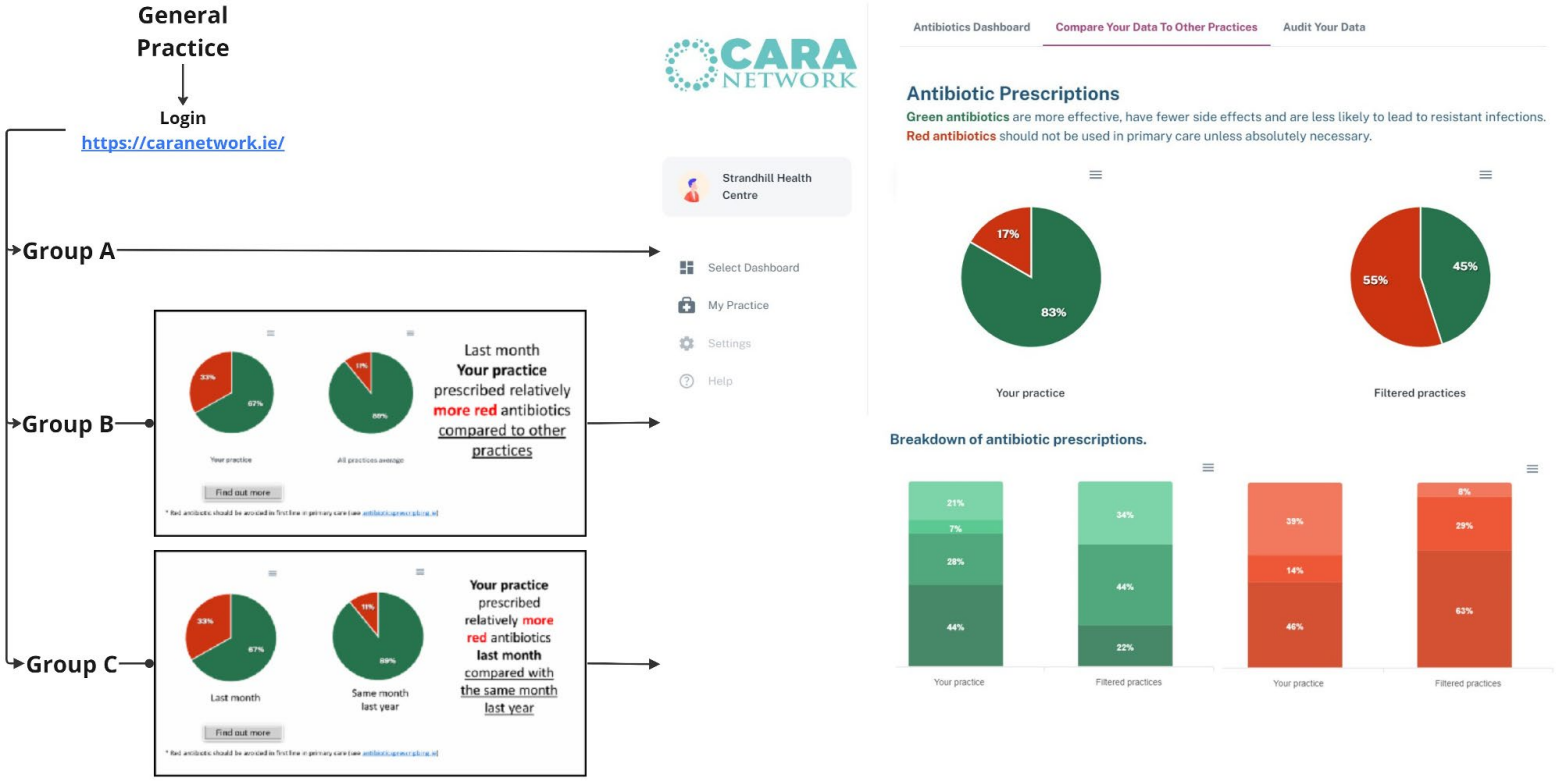
Overview of the allocation of practices to group A, B or C

General practices will be randomised to one of three different initial landing pages (groups): the full dashboard or one of two key messages. All practices have access to the same dashboards and view identical information but two groups will be shown a key message before they can continue to the full CARA dashboard [27]. The key messages are similar and focus on one of the charts from the existing dashboard (Figs. 1 and 2):

### Participants

General practices that register with the CARA network for access to the dashboards will be randomised (practice level) using a computer-generated system, which will allocate to either group A, B or C using 1:1:1 randomisation (Fig. 1). Practices are NOT allocated to different dashboards but to different initial landing pages from where they can access the full dashboard.

### Recruitment

General practices will be invited to join the CARA network [18] through social media and direct mailing through the Irish College of General Practitioners. Registration with the CARA network [18] is open to all general practices. Once registered and after confirming terms and conditions, the general practice receives a link to CARAconnect (to de-identify, extract and upload data) and view their practice data in the CARA dashboards.

### Outcomes

The primary outcome is behavioural engagement (measured as the number of ‘clicks’, ‘viewings’ and ‘sessions’) with the CARA dashboard after one month of dashboard usage collected through Google Analytics (Table 1).

**Table 1:** Terminology definition of behavioural engagement outcomes (from Google Analytics [28])

Outcomes	Definition
Conversion event	Every ‘click’ on the antibiotic dashboard page (clicks include filters, comparisons with other practices and audit)
Session Conversion rate	The number of conversion events (at least one) divided by the total number of sessions*
Engaged sessions	The number of sessions* that lasted at least 10 s, had 1 or more conversion events or 2 or more page or screen views
Engagement rate	The number of engaged sessions divided by the total number of sessions*
Bounce rate	The number of sessions that were not engaged divided by the total number of sessions*
Average session duration	The average duration (in seconds) of users’ sessions*.

* A session initiates when a user opens the CARA dashboard. By default, a session ends or times out after 30 min of user inactivity

### Sample size

Sample size is calculated based on the conversion event (number of clicks on the interaction with the antibiotic dashboard page, see Table 1) using a comparative usability test to determine differences between group A, B and C (between-group design) [29, 30]. A comparative usability test is a method for describing and comparing the usability of more than one application [29, 30]. The variance estimation is obtained from a usability test for a similar experiment, which reported the mean number of mouse clicks for dashboard usage (mean = 23.6 and standard deviation = 14.8) [31]. A total sample size of 87 users or practices (29 per group) is estimated to be sufficient to observe a 10% difference [7, 32] with 5% statistical confidence [29, 30].

### Data collection

This study will run for a limited time, dashboard usage data will be collected for each practice for a month from the time of enrolment. All practices registering with the CARA network will be included in the randomisation. Dashboard usage data will be collected through Google analytics, which is an integrated part of CARA. Google Analytics provides anonymous data on the behavioural engagement with a page irrespective of who is engaging. Therefore, three identical websites (dashboards) will be set up, two of which will contain an additional landing page with a key message. At no stage IP addresses will be recorded or tracked. Table 1 shows the behavioural engagement data that will be collected.

### Data analysis

The quantitative information collected will be summarised (descriptive characteristic table) using the mean and standard deviation or, if not normal, with medians and interquartile ranges. Categorical variables will be summarised with counts and proportions. Depending on the data distribution, the continuous measures analysis will use linear regressions or generalised estimating equations for binary and count data. The *N* − 1 two-proportion test (A/B testing) will be used (for small sample sizes) to compare differences in proportions [33]. All statistical tests will be 2-tailed and significance will be set at a p-value of 0,05. Analysis will be performed using R-4.0.3® software.

## Discussion

This paper outlines the protocol to explore how different initial key messages impact the level of behavioural engagement with the CARA dashboard. User research can determine a dashboard’s overall performance and ensures rigorous design and implementation, assesses usefulness, operability, ease of use, satisfaction, user interface, content, as well as system capabilities [11, 34].

Even though similar dashboards on prescribing that include user involvement, have been developed in Canada and the United Kingdom, no users’ involvement process or user research evaluation were described [35, 36]. User research is a process that should continue after the dashboard is launched and includes a process evaluation in a real-world setting [11, 30, 37]. However, process evaluations are still rarely used despite the proliferation of dashboards [4, 6]. For instance, Fazaeli S et al. designed and implemented a COVID-19 management dashboard, used usability testing to examine users’ satisfaction and incorporated feedback into the dashboard design [36]. However, they did not evaluate the complete user research process [10].

In general, little evidence is available on the evaluation of dashboard engagement or dashboard usage data analysis [7, 8, 38]. User engagement is not static and is a prolonged process which varies over time. Its measurement reflects the degree of involvement a user has with the dashboard or the system [39, 40]. User engagement measures quantify usability, but a recent systematic review only identified one study that recorded dashboard engagement [7]. It was unclear if any of the other existing dashboards developed for use in primary care and included in the review, went through a usability evaluation process [7].

However, the application of behavioural engagement and its metrics during the development and implementation of dashboards needs to be explored to know how the user interacts with the dashboard, how they make sense of the data presented in the dashboard, how effective and meaningful the dashboard design is and the benefit gained from using this dashboard and data [41]. Furthermore, engaging with dashboard users reveals interesting challenges that may be addressed through dashboard design and implementation. For instance, peer performance visualisation is often used to promote dashboard engagement and motivation [42].

The CARA infrastructure will be implemented and tested in an incremental number of general practices. To support the continued use and sustainability of the CARA dashboards, their performance and engagement will be monitored to regularly implement updates and improvements. The implications of this study will improve our understanding of how users interact with the CARA dashboard, as well as how different initial key messages impact behavioural engagement within the CARA dashboard. Building on this research, inclusion of key performance indicators for evaluating, measuring and improving prescribing will be a follow on study for CARA.

## Data Availability

No datasets were generated or analysed during the current study.

## Abbreviations

ADR: Action Design Research
CARA: Collaborate, Analyse, Research, Audit
PMS: Patient Management System
